# Peripheral blood microR-146a and microR-29c expression in children with *Mycoplasma pneumoniae* pneumonia and its clinical value

**DOI:** 10.1186/s13052-023-01500-0

**Published:** 2023-09-13

**Authors:** Jingcai Wang, Chunyan Guo, Lixin Yang, Peng Sun, Xiaoqing Jing

**Affiliations:** Department of Pediatric Medicine, Affiliated Hospital of Chengde Medical College, Chengde, 067000 China

**Keywords:** miR-29c, Mir-146a, *Mycoplasma pneumoniae* pneumonia, Inflammatory factors, diagnostic biomarker

## Abstract

**Background:**

We investigated changes in microR-29c and microR-146a expression in the serum of children with *Mycoplasma pneumoniae* pneumonia, analysed their relationship with inflammatory factors and disease severity, and evaluated their diagnostic significance.

**Methods:**

Fifty-six children with *Mycoplasma pneumoniae* pneumonia were enrolled as the *Mycoplasma pneumoniae* pneumonia group; 37 healthy children were enrolled as the control group. The microR-29c or microR-146a serum expression levels were determined using real-time quantitative reverse transcription polymerase chain reaction. Interleukin-17, tumour necrosis factor-alpha, and interleukin-1 beta levels were detected using enzyme-linked immunosorbent assay. The correlation between serum microR-29c or microR-146a expression and inflammatory factors was analysed using the Pearson’s method. Receiver operating characteristic curves were used to evaluate the diagnostic value of serum microR-29c, microR-146a, and their combined detection in *Mycoplasma pneumoniae* pneumonia.

**Results:**

Compared with that in healthy children, the microR-29c and microR-146a serum levels were significantly downregulated in children with *Mycoplasma pneumoniae* pneumonia; the decrease was more obvious in children with severe cases than that in those with mild cases. In addition, microR-29c and microR-146a were negatively correlated with increased expression of interleukin-17, tumour necrosis factor-alpha, and interleukin-1 beta. Receiver operating characteristic curves showed that a combination of microR-29c and microR-146a was highly suitable for diagnosing *Mycoplasma pneumoniae* pneumonia.

**Conclusion:**

Serum microR-29c and microR-146a were underexpressed in children with *Mycoplasma pneumoniae* pneumonia, and diagnostic accuracy was significantly improved with combined microR-29c and microR-146a detection. Therefore, both microR-29c and microR-146a levels can be used as biomarkers for the diagnosis of *Mycoplasma pneumoniae* pneumonia.

## Introduction

*Mycoplasma pneumoniae* pneumonia (MPP) is one of the most common causes of respiratory infections in children [1]. It can invade the lungs and other tissues and cause upper and lower respiratory infections in children [2]. MMP is a relatively common respiratory infectious disease observed clinically. The clinical manifestations include cough, fever, anorexia, and headache; however, most cases of MMP have atypical symptoms [3]. MPP causes 20–40% of community-acquired pneumonia in children, and the incidence is even higher during epidemics [4]. Severe MPP in children is often accompanied by lung necrosis, atelectasis, lung consolidation, and respiratory failure; MPP has led to increased morbidity, mortality, and medical costs in China [5]. Due to the insidious onset and atypical clinical symptoms of early MPP, the development of severe MPP will continue to threaten the health of children and bring economic burden to affected societies. Therefore, it is particularly important to explore a scheme or means for early treatment intervention or diagnosis of MPP.

As natural gene regulators, micro (mi)-RNAs regulate target genes at the post-transcriptional level and are widely involved in various biological behaviours, such as cell metabolism, proliferation, differentiation, and apoptosis. These gene regulators have a regulatory role in the development of inflammatory diseases [6, 7]. Abnormally expressed miRNAs can predict the occurrence and development of biological diseases and are closely involved in regulating important biological cell behaviours [8, 9]. MicroR-29c, a member of the miR-29 family, plays a crucial role in children with MPP and may be a novel target for the prevention and treatment of MPP and a potential novel biomarker for the diagnosis of MPP [10]. In addition, miRNA-146a, an miRNA closely related to inflammation, has been verified to play a vital role in immunity, tumour development, and inflammation [11, 12]. Studies have shown that the expression of miR-146a is inhibited in patients with refractory MPP and that miR-146a is a potential target for regulating inflammation in MPP [13]. However, little research has been conducted on the expression of miR-146a and miR-29c in the peripheral blood of children with MPP.

Therefore, in this study, the expression of miR-146a and miR-29c in the peripheral blood of children with MPP and healthy controls was evaluated and compared. Furthermore, the correlation between the expression and the clinical indices of MPP was analysed to detect the clinical value of miR-29c and miR-146a in peripheral blood.

## Materials and methods

### Research participants

A total of 56 patients (25 males, 31 females; mean age, 7.78 ± 2.91 years) with MPP from Affiliated Hospital of Chengde Medical College between January 2019 and December 2021 were enrolled and categorized as the MPP group. Enrolled patients were further categorized into a severe group (*n* = 25) and a mild group (*n* = 31). In addition, 37 healthy individuals (18 males, 19 females; mean age, 28.59 ± 6.81 years) in our hospital over the same period were enrolled and assigned as the healthy group. The inclusion criteria for the MPP group were as follows: (1) age ranging from 1 to 18 years; (2) meeting the diagnostic criteria of *Mycoplasma pneumoniae* pneumonia: serum *Mycoplasma pneumoniae* immunoglobulin M (IgM) antibody positive and titres ≥ 1:80 (using Japan Celadia-Micco II *Mycoplasma pneumoniae* antibody detection kit and passive agglutination detection). *Mycoplasma* infection was confirmed by bacterial culture of serum or sputum secretion and drug sensitivity tests. Other types of microbial infections, such as *Chlamydia pneumoniae*, *Streptococcus pneumoniae*, *Staphylococcus*, and *Adenovirus*, were excluded; and (3) complete clinical data. The exclusion criteria were as follows: (1) antibiotic or hormone therapy taken before admission and (2) concurrent infection of other sites or damage to liver and kidney function. The study was performed in accordance with the ethical standards of the responsible committee on human experimentation (Ethics Committee of Affiliated Hospital of Chengde Medical College; approval code, CYFYLL2022499) and with the Helsinki Declaration of 1975, as revised in 2008. All patients or their families were informed of the study, agreed to participate, and signed informed consent.

### Detection of expression levels of miR-146a and miR-29c in MPP through real-time quantitative reverse transcription polymerase chain reaction (qRT-PCR)

Patient serum was collected within 24 h of admission before treatment peripheral blood and immediately stored at -80 ℃ until use. Total RNA was extracted using the TRIzol kit. DNA was reverse-transcribed into complementary DNA using the PrimeScript 1st Strand cDNA synthesis kit (Takara, Dalian, China). qRT-PCR was performed using the ABI7300 real-time PCR system (Applied Biosystems, Foster City, CA, USA) and the SYBR®Premix Ex Taq™Kit (Takara). Relative gene expression was evaluated using 2^−ΔΔCt^; glyceraldehyde 3-phosphate dehydrogenase was used as an endogenous control. The conditions were as follows: 95 °C for 2 min, 10 cycles of 95 °C for 45 s and 55 °C for 60 s, followed by 30 cycles of 95 °C for 30 s and 55 °C for 45 s. The primer sequences used are listed in Table 1.

**Table 1:** qRT-PCR primer information

Primer name	Sequences
miR-146a forward	5′- GTGCAGGGTCCGAGGT-3′
miR-146a reverse	5′- CAACACCAGTCGATGGGCTGT-3′
miR-29c forward	5′-CTGACCTTAGCACCATTTGAAATC-3′
miR-29c reverse	5′-TATCGTTGTACTCCACTCCTTGAC -3′
GAPDH forward	5′-GGAGCGAGATCCCTCCAAAAT-3′
GAPDH reverse	5′-GGCTGTTGTCATACTTCTCATGG-3′

*Abbreviations*: *qRT-PCR* Real-time quantitative reverse transcription-polymerase chain reaction, *mi* Micro, *GADPH* Glyceraldehyde 3-phosphate dehydrogenase

### Enzyme-linked immunosorbent assay (ELISA)

The concentrations of interleukin-17 (IL-17), tumour necrosis factor-alpha (TNF-α), and interleukin-1 beta (IL-1β) in the serum were detected using commercial ELISA kits (Nanjing Jiancheng Bioengineering Institute, Nanjing, China) according to the manufacturer's instructions [14]. Briefly, the blank, standard, and sample wells were prepared. After washing, 100 μl of enzymatic secondary antibody was added and incubated at 37 ℃ for 60 min. After a repeat washing, chromogenic solution was added; the solution was then incubated at room temperature for 20 min, and the termination solution was added. The absorbance at 450 nm of each well was measured using the colour intensity development. The same methods as those described above were used for sampling and analysing the serum in the healthy control group.

### CRP, PCT and LDH

Blood samples were taken after overnight fasting for the determination of C-reactive protein (CRP), procalcitonin (PCT), and lactate dehydrogenase (LDH). Blood sample analysis is performed in the hospital's central laboratory using commercially available kits commonly used in hospital clinical practice.

### Statistical analysis

All experiments were performed thrice. The data are expressed as mean ± standard deviation (SD). Data analysis was performed using SPSS 21.0 (SPSS, Inc., Chicago, IL, USA) and GraphPad Prism 8.0 software (GraphPad Software, Inc., La Jolla, CA, USA). Comparisons between the two groups were analysed using Student’s t-test. The correlations among indices were analysed using the Pearson correlation coefficient. The diagnostic value of miR-29c, miR-146a, or their combination was evaluated using a receiver operating characteristic (ROC) curve. *P* value < 0.05 was considered statistically significant.

## Results

### Underexpression of miR-29c and miR-146a in children with MPP

**Fig. 1:**
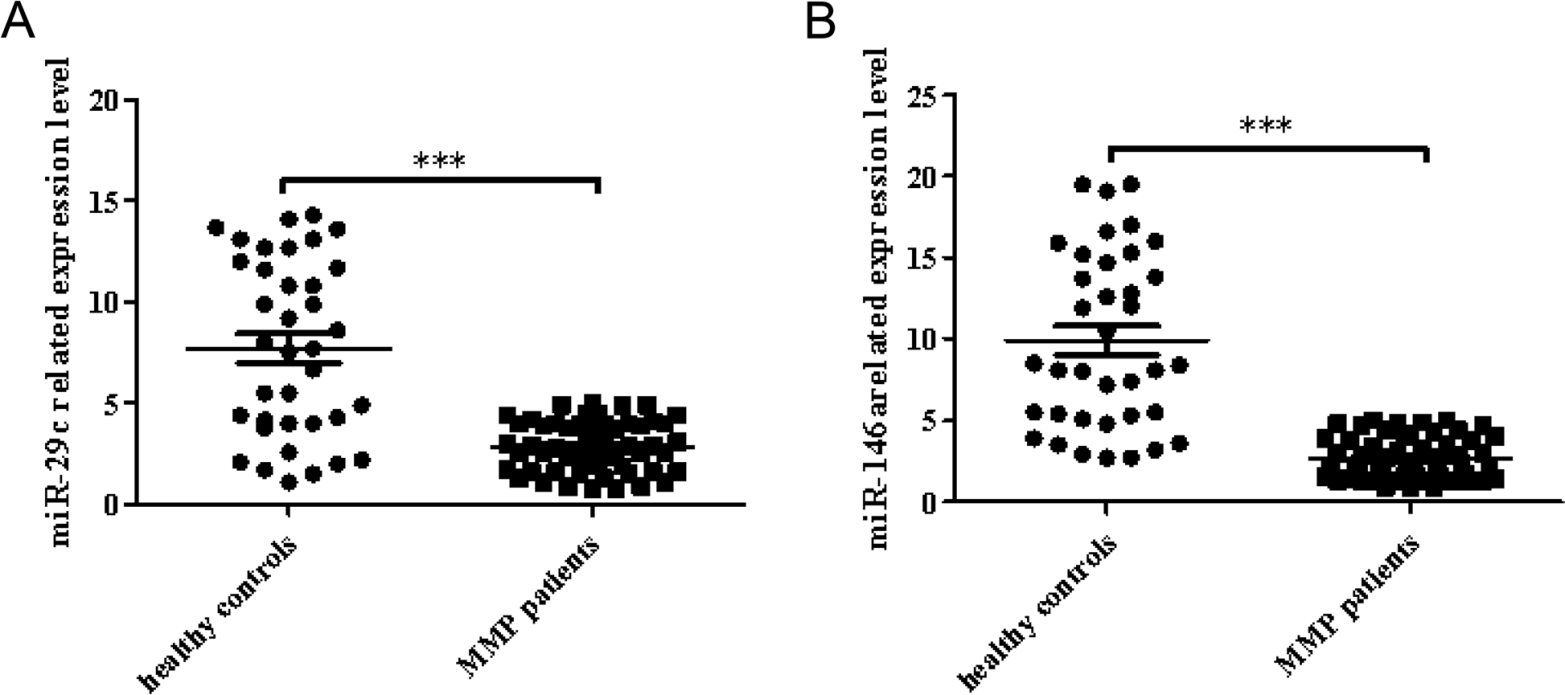
Underexpression of miR-29c and miR-146a in children with MPP. **A** miR-29c, (**B**) miR-146a (***P < 0.05: MMP group vs. healthy group). Abbreviations: mi, micro; MMP, Mycoplasma pneumoniae pneumonia

The qRT-PCR results showed that the expression levels of miR-29c and miR-146a in patients with MPP were significantly decreased compared to the healthy controls (Fig. 1, *P* < 0.05).

### Expression levels of inflammatory factors in children with MPP

**Fig. 2:**
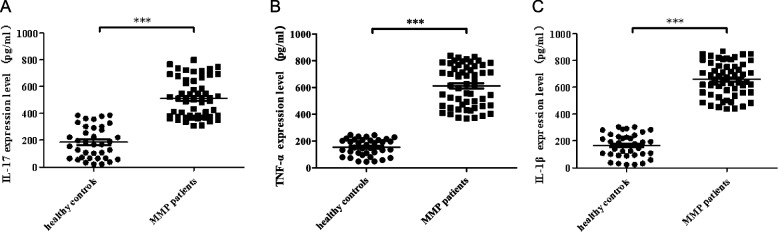
Increased expression of inflammatory cytokines in children with MPP. **A** IL-17, (**B**) TNF-α, (C) IL-1β (***P < 0.05: MMP group vs. healthy group). Abbreviations: IL, interleukin; MMP, Mycoplasma pneumoniae pneumonia; TNF- α, tumour necrosis factor-alpha; β, beta

The expression levels of IL-17, TNF-α, and IL-1β in children with MPP were determined using ELISA. The results showed that the levels of IL-17, TNF-α, and IL-1β in children with MPP were significantly higher than those in the control group (Fig. 2, *P* < 0.05).

### Expression levels of CRP, PCT and LDH in children with MPP

**Fig. 3:**
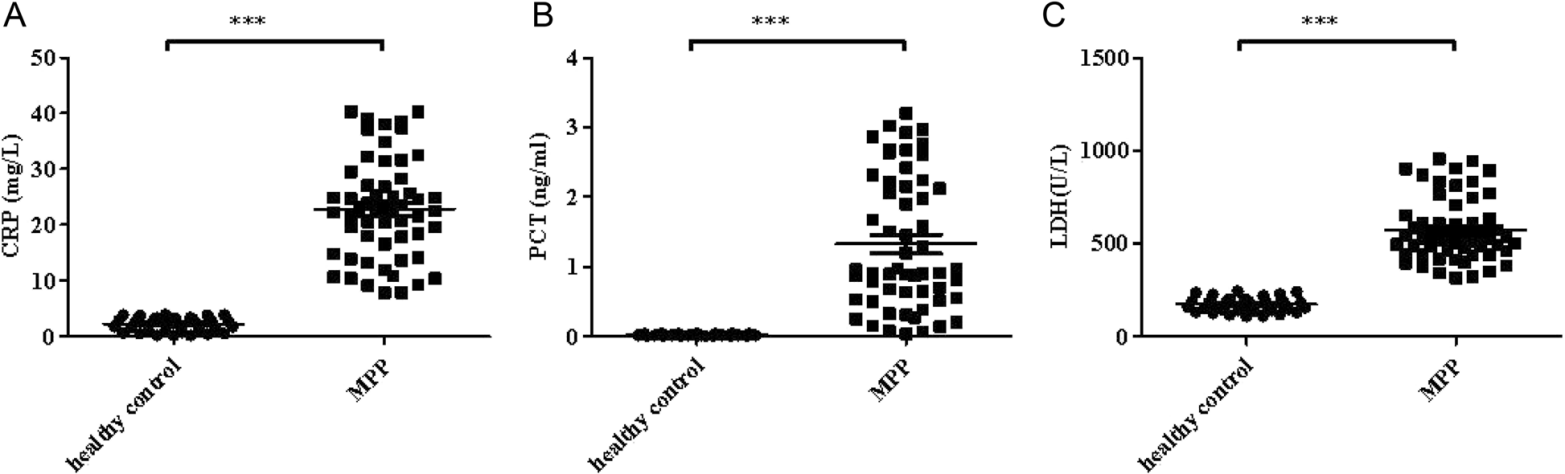
Increased expression of CRP, PCT and LDH in children with MPP. **A** CRP, (**B**) PCT, (**C**) LDH (***P < 0.05: MMP group vs. healthy group). Abbreviations: CRP, C-reactive protein; PCT, procalcitonin; LDH, lactate dehydrogenase; MMP, Mycoplasma pneumoniae pneumonia

We also detected the expression levels of CRP, PCT and LDH in MPP children. The results showed that CRP, PCT, and LDH levels in MPP children were significantly higher than those in control group (Fig. 3, *P* < 0.05).

### MiR-29c and miR-146a correlated with inflammatory factors in children with MPP

**Fig. 4:**
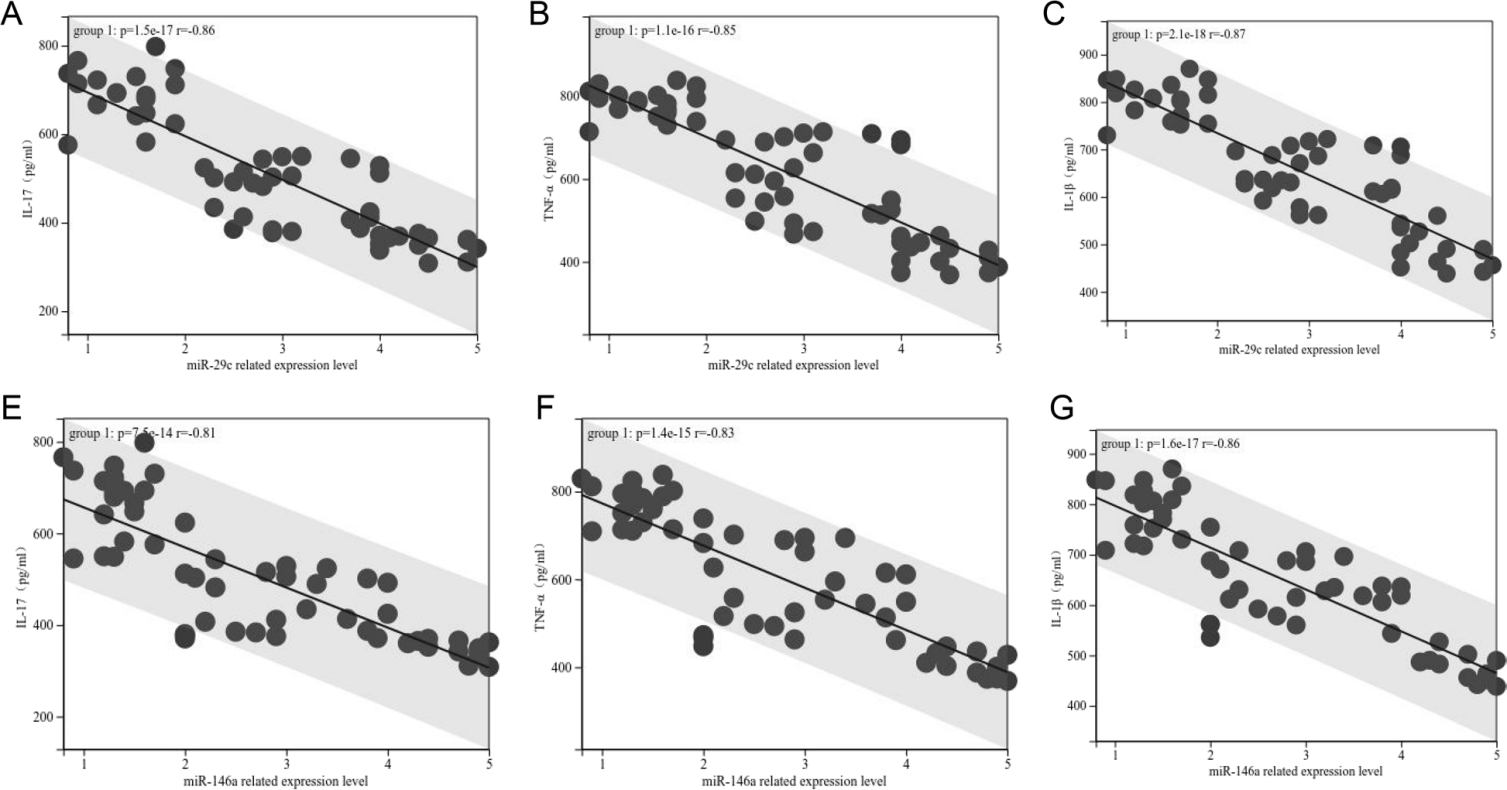
Correlation between the expression of miR-29c and miR-146a and inflammatory factors in serum of children with MPP. **A** Expression of miR-29c negatively correlated with IL-17 (*r* = 0.86, *P* < 0.001). **B** Expression of miR-29c negatively correlated with TNF-α (*r* = 0.85, *P* < 0.001). **C** Expression of miR-29c negatively correlated with IL-1β (*r* = 0.87, *P* < 0.001). **D** Expression of miR-146a negatively correlated with IL-17 (*r* = 0.81, *P* < 0.001). **E** Expression of miR-146a negatively correlated with TNF-α (*r* = 0.83, *P* < 0.001). **F** Expression of miR-146a negatively correlated with IL-1β (*r* = 0.86, *P* < 0.001). Abbreviations: mi, micro; IL, interleukin; TNF- α, tumour necrosis factor-alpha; β, beta

Pearson’s correlation coefficient was used to analyse the correlation between the expression levels of miR-29c and miR-146a and inflammatory factors in children with MPP. The results showed that the relative expressions of miR-29c and miR-146a negatively correlated with the levels of IL-17, TNF-α, and IL-1β (Fig. 4, *P* < 0.05).

### MiR-29c and miR-146a correlated with CRP, PCT and LDH in children with MPP

**Fig. 5:**
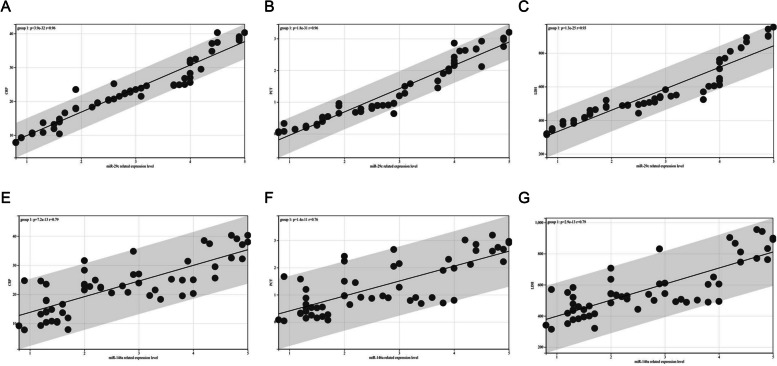
Correlation between the expression of miR-29c and miR-146a and CRP, PCT and LDH in serum of children with MPP. **A** Expression of miR-29c negatively correlated with CRP (*r* = 0.96, *P* < 0.001). **B** Expression of miR-29c negatively correlated with PCT (*r* = 0.96, *P* < 0.001). **C** Expression of miR-29c negatively correlated with LDH (*r* = 0.93, *P* < 0.001). **D** Expression of miR-146a negatively correlated with CRP (*r* = 0.79, *P* < 0.001). **E** Expression of miR-146a negatively correlated with PCT (*r* = 0.76, *P* < 0.001). **F** Expression of miR-146a negatively correlated with IL-1β (*r* = 0.79, *P* < 0.001). Abbreviations: mi, micro; CRP, C-reactive protein; PCT, procalcitonin; LDH, lactate dehydrogenase; MMP, Mycoplasma pneumoniae pneumonia

Pearson’s correlation coefficient showed that the relative expression levels of miR-29c and miR-146a were also negatively correlated with CRP, PCT and LDH (Fig. 5, *P* < 0.05).

### Evaluation of clinical value of miR-29c and miR-146a in diagnosing MPP

**Fig. 6:**
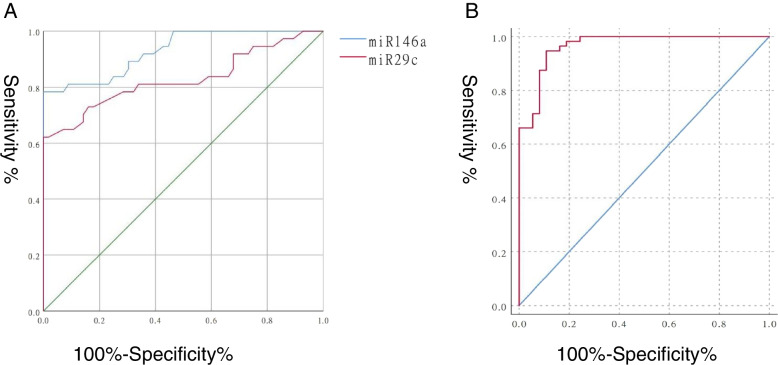
Evaluation of clinical value of miR-29c and miR-146a in the diagnosis of MPP. **A** ROC curve based on miR-29c or miR-146a expression. **B** ROC curve based on miR-29c + miR-146a expression. Abbreviations: MPP, Mycoplasma pneumoniae pneumonia; mi, micro; ROC, receiver operating characteristic

To evaluate the diagnostic value of miR-29c and miR-146a in children with MPP, ROC curves were generated based on the serum expression levels of these molecules. The results showed that both miR-29c and miR-146a had high diagnostic accuracy, with an area under the curve (AUC) of 0.828 (sensitivity: 62.2%; specificity: 98.2%) and 0.930 (sensitivity: 78.4%; specificity: 96.4%), respectively (Fig. 6A and B). Moreover, miR-29c combined with miR-146a had the best diagnostic value (AUC = 0.966, sensitivity: 94.6%; specificity: 89.2%; Fig. 6C), indicating that this combination led to a significant improvement in diagnostic ability.

### Expression levels of miR-29c and miR-146a in severe or mild MPP

**Fig. 7:**
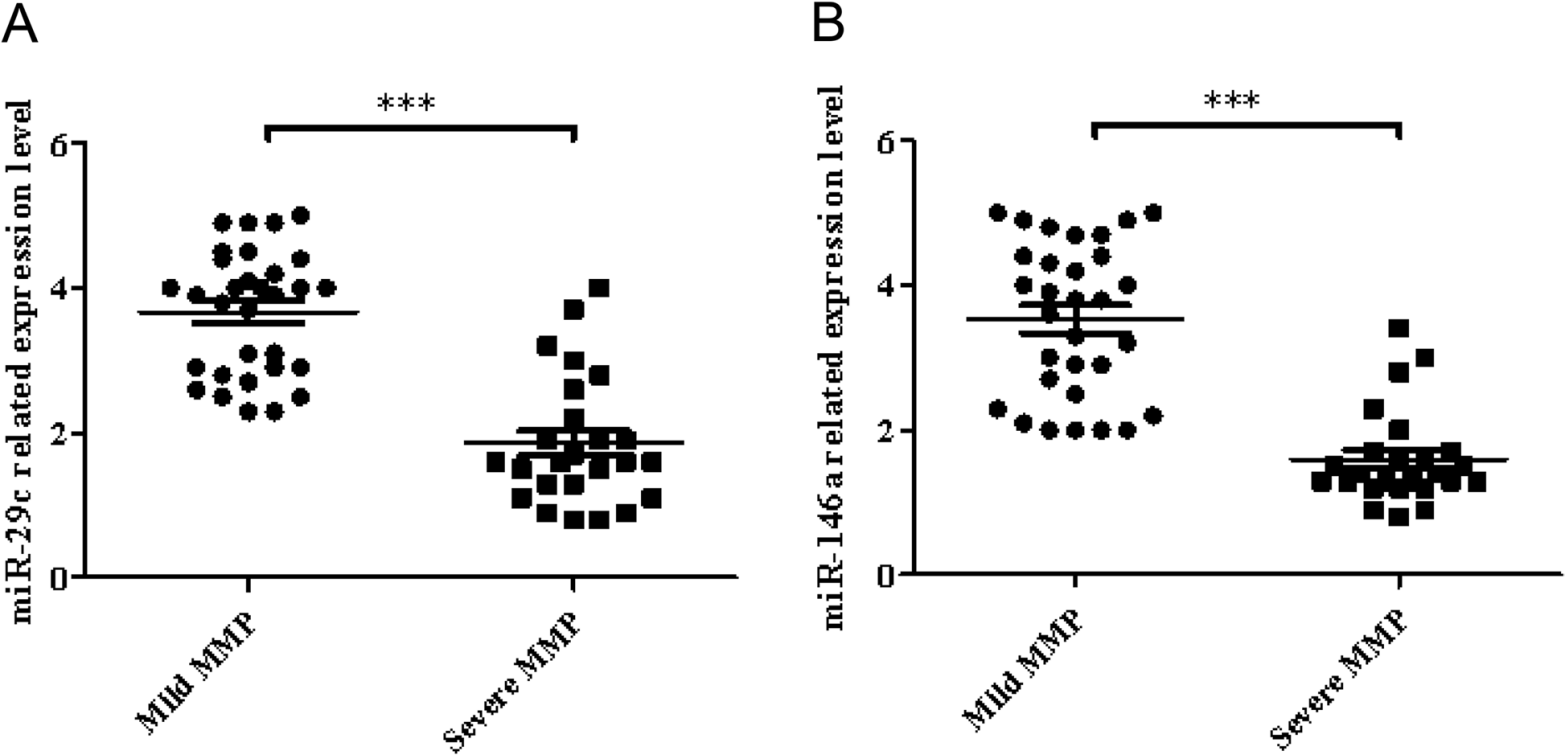
Lower serum miR-29c and miR-146a expression in children with severe MPP. Both miR-29c and miR-146a showed lower expression levels in children with severe MPP. **A** miR-29c, (**B**) miR-146a (***P < 0.05: severe MPP vs. mild MPP). Abbreviations: MPP, Mycoplasma pneumoniae pneumonia; mi, micro

Furthermore, we compared the expression levels of miR-29c and miR-146a in 25 children with severe MPP and 31 children with mild MPP and found that the levels of miR-29c and miR-146a in children with severe MPP were lower than those in children with mild disease (Fig. 7, *P* < 0.05).

## Discussion

MPP can lead to myocarditis or nephritis and considerably threaten the quality of life and safety of children. If not treated promptly, MPP can damage multiple organs and even lead to death [15]. Studies have shown that MP-IgM has an acceptable specificity for the diagnosis of MPP [16]. MP-IgM appears in the first week after infection with Mycoplasma pneumoniae and peaks in the third week after infection [16]. However, MP-IgM detection is mainly based on ELISA, which has low sensitivity and has not become a reference standard and diagnostic standard for MPP diagnosis. Therefore, searching for molecular markers with high sensitivity and specificity for the early diagnosis of MPP is of great clinical significance. miRNAs are important multifunctional regulators that participate in cell differentiation, proliferation, and apoptosis by affecting signal transduction [17, 18]. Abnormal expression of miRNA is closely related to the occurrence of diseases [19, 20]. In this study, we first evaluated the diagnostic role of two miRNAs, miR-29c and miR-146a, in MPP. The analyses demonstrated that the expression levels of miR-29c and miR-146a were both downregulated in children with MPP. Furthermore, we evaluated the expression levels of miR-29c and miR-146a in severe and mild MPP and found that the expression levels of miR-29c and miR-146a were lower in severe MPP than those in mild MPP.

MicroR-29c, a member of the miR-29 family, has been found to be expressed at low levels in the sera of patients with Parkinson’s disease (PD) and is involved in inflammatory activation. Thus, miR-29c may have potential as a PD biomarker [21, 22]. In addition, miR-29c plays an important role in children with MPP by regulating excessive inflammation [10]. In this study, the serum level of miR-29c in children with MPP was significantly lower than that in the control group, confirming the role of miR-29c in the occurrence and development of MPP. IL-17 is an early initiating factor of the T-cell-induced inflammatory response. As a factor that promotes inflammation, IL-17 stimulates inflammatory tissues by inducing bronchial epithelial cells and fibroblasts [23]. Some studies have found abnormal expression of IL-17 in patients with MPP, suggesting that IL-17 has an important role in MPP; however, the specific mechanism remains unclear [5]. Serum IL-17 levels in children with MPP were significantly increased and negatively correlated with miR-29c expression in the present study, suggesting that miR-29c and IL-17 may be involved in MPP development. Moreover, we found that the expression of TNF-α, IL-1β, CRP, PCT and LDH was upregulated in the serum of children with MPP and negatively correlated with miR-29c expression. In the development of some diseases, the downregulation of miR-29c expression has been reported to promote the secretion of pro-inflammatory cytokines and activate the NLRP3 inflammasome and nuclear factor-kappa-light-chain-enhancer of activated B cells, thereby upregulating the expression of IL-1β [20]. However, the potential involvement of miR-29c in the expression of TNF-α and IL-1β in MPP and the mechanisms of regulation need to be further analysed. The ROC curve analysis showed that the AUC of serum miR-29c in diagnosing MPP was 0.828; the sensitivity was 62.2% and the specificity was 98.2%. Taken together, these results suggest that miR-29c may become a novel target for the prevention and treatment of MPP and is a potential biomarker for the assessment of prognosis.

MicroR-146a expression has been found to be reduced in patients with MPP [13], which is consistent with the results of the current study. In addition, we found that miR-146a was negatively correlated with the expression of inflammatory factors in children with MPP, suggesting that miR-146a may be involved in the inflammatory response. This hypothesis could be further analysed and confirmed in future experiments. In addition, the ROC curve of miR-146a in the diagnosis of MPP showed that the AUC, sensitivity, and specificity of miR-146a in the diagnosis of MPP were 0.930, 78.4%, and 96.4%, respectively, indicating that miR-146a had value in diagnosing MPP. Further, we found that although the serum level detection of miR-29c or miR-146a had value in diagnosing MPP, the combined detection of the two markers was superior to single detection, and the analysis results showed that the combined detection of miR-29c and miR-146a can increase the sensitivity and AUC.

The results of this study all indicate that miR-29c and miR-146a may be involved in the development of MPP, and they are expected to become potential targets for the early diagnosis of MPP. In addition, miR-29c and miR-146a were significantly reduced in patients with severe MPP compared to patients with mild MPP. These results may also suggest that reduced expression of miR-29c and miR-146a in serum is a valuable biomarker for patients with severe MPP and could help distinguish between severe and mild MPP in early diagnosis. However, the present study also has some limitations. First, the sample size of this study is limited and may not be representative of all MPP patients. Second, there are no in vitro experiments to confirm the underlying mechanisms of miR-29c and miR-146a in MPP. Therefore, we hope to further expand the test sample in future experiments and detect more about the role of miR-29c and miR-146a in MPP through in vitro experiments.

## Conclusions

In conclusion, miR-29c and miR-146a were both expressed at low levels in the serum of children with MPP, and their expression was negatively correlated with the severity of MPP and the level of inflammatory factors. These results suggested that reduced levels of miR-29c and miR-146a are associated with abnormal inflammatory responses and severe and mild disease in MPP children. Therefore, the findings of this study indicate that the detection of miRNAs in serum has broad prospects for improving the diagnosis of MPP and may provide a basis for a highly comprehensive understanding of the pathogenesis of MPP.

## Data Availability

All data generated or analysed during this study are available from the. corresponding author on reasonable request.

## Abbreviations

MPP: *Mycoplasma pneumoniae* Pneumonia
PD: Parkinson’s disease
